# Formalin Treatment of Bed Sores

**Published:** 1919-08-23

**Authors:** 


### FORMALIN TREATMENT OF BED SORES.

THERE are many generally accepted measures for dealing with bed sores—for example, cleanliness, frequent change of position, and special air- or water-beds—and also a great number of relatively efficient local applications. To add another to the list may seem, perhaps, unnecessary, but, in view of the highly successful result claimed for the use of dilute formalin in several American hospitals, we find excuse for outlining this method. The requirements are mainly that the application should penetrate, disinfect, deodorise, check secretion, and have some constricting action on the vessels. Bichloride of mercury and carbolic fulfil some of these at the expense of others, and theoretically they find formalin approximates closely to the ideal.

It is used in a solution made up from 1 part of the usual 40 per cent. formalin in 200 parts of water, and it is stated, when employed with discretion, does not show any irritative action. At the same time, when applied by a syringe, it is possible to reach all the ramifications and crypts of an ulcer, while, although a number of such dressings may have to be done on various patients, the dresser's hands are readily protected from the cumulative exiccating or irritant effect by simple rubber gloves. A satisfactory routine has been to use preliminary plain warm boiled water, followed by *cold* formalin, alternating several times and thus including the stimulating effects of a changing temperature.

In the intervals between these dressings it has been found that subiodide of bismuth, dusted everywhere over the raw area as a moderately thick absorbent and antiseptic layer, gave the best results. At the outset the whole procedure must be repeated several times a day, but later the frequency can be greatly reduced as the crusts become cleaner and drier, and may be left untouched while cicatrisation takes place. Although this method is largely opposed to our accepted experiences with regard to the action of formalin on raw-skin surfaces, success has apparently been obtained, but we would reserve judgment until a fuller trial has been given.

